# Prevalence of condomless anal intercourse and recent HIV testing and their associated factors among men who have sex with men in Hangzhou, China: A respondent-driven sampling survey

**DOI:** 10.1371/journal.pone.0167730

**Published:** 2017-03-08

**Authors:** Runhua Li, Hui Wang, Xiaohong Pan, Qiaoqin Ma, Lin Chen, Xin Zhou, Tingting Jiang, Lin He, Junfang Chen, Xingliang Zhang, Yan Luo, Shengjun Xi, Xin Lv, Shichang Xia

**Affiliations:** 1 Department of HIV/AIDS and STDs Control & Prevention, Zhejiang Provincial Center for Disease Control and Prevention, Hangzhou, Zhejiang Province, People's Republic of China; 2 Department of Epidemiology and Health Statistics, School of Medicine, Ningbo University, Ningbo, Zhejiang Province, People's Republic of China; 3 Center for Disease Control and Prevention of Hangzhou City, Hangzhou, Zhejiang Province, People's Republic of China; 4 Center for Disease Control and Prevention of Xiacheng District, Hangzhou, Zhejiang Province, People's Republic of China; UCSF, UNITED STATES

## Abstract

Men who have sex with men (MSM) are a large high-risk population for HIV infection in recent years in China. A cross-sectional survey was conducted in Hangzhou, China, to determine rates of condomless anal intercourse (CAI), recent HIV testing (in the recent year) and associated factors using respondent-driven sampling. Questionnaires using face-to-face interviews were employed to collect data on sexual risk behaviors and HIV testing. Five hundred eleven MSM were recruited, of which 459 (89.8%) had anal intercourse in the past 6 months. Of these 459 participants, 457 (99.6%) answered whether they had taken an HIV test in the recent year, so only their data were analyzed. Weighted data were analyzed using bivariate and multivariate logistic regression analysis. The CAI rate with male partners in the past 6 months was 43.7% (95% confidence interval [CI], 34.0–51.5%), while the rate of condomless vaginal intercourse (CVI) was 21.6% (95% CI, 15.6–32.3%). The prevalence of recent HIV testing was 56.8% (95% CI, 48.7–66.5%), while the prevalence of HIV and syphilis were 8.8% and 6.5%, respectively. Multivariate analysis indicated that CAI was associated with earlier homosexual debut, suicidal inclinations, childhood sexual abuse, HIV testing in the recent year, and lower estimate of HIV prevalence. Recent HIV testing was associated with homosexual debut age, engaging in CAI with male partners in the past 6 months, having oral sex in the past 6 months, self-perceived higher likelihood of HIV infection, knowing about antiretroviral therapy for HIV/AIDS, receiving AIDS/sexually transmitted infection (STI) interventions in the past year, and syphilis infection. Given high prevalence of HIV and syphilis, high levels of CAI and CVI, and low HIV testing rate, the results indicated high risk of HIV infection and transmission among MSM. HIV prevention interventions should target MSM with early homosexual debut and psychosocial health problems, while HIV/AIDS education among MSM should focus on increasing knowledge of HIV risk, estimated HIV prevalence and antiretroviral therapy, and improving risk perception of HIV acquisition.

## Introduction

Men who have sex with men (MSM) have been a large high-risk population for human immunodeficiency virus (HIV) infection and transmission because of their more hidden and stigmatized nature [1, 2], more diverse sexual networks [3, 4], and tendency toward risky sexual behaviors such as multiple sexual partners and condomless anal intercourse (CAI) [5]. The proportion of homosexual transmission among annually reported HIV-infected cases has rapidly increased in the last decade, from 2.5% in 2006 to 25.8% in 2014. Furthermore, the national HIV prevalence among MSM reached 7.7% in 2014 in China, according to the 2015 China AIDS Response Progress Report [6].

Given these figures, promoting condom use as a means of preventing sexually transmitted infection (STI) and reducing the number of new HIV infections among MSM has become a public health priority, and is a critical element of prevention programs globally [7–9]. The risk of HIV transmission during CAI is approximately 18 times than that of transmission during condomless vaginal intercourse (CVI) [10–12], and consistent condom use has been found to reduce this risk by 70% [8]. Anal intercourse increases the risk of HIV transmission due to injuries to the thin anal and fragile rectal mucosa, which is lined by a large number of HIV target cells such as CD4+ cells. CAI is more likely to cause trauma, and without a barrier, the probability of HIV transmission is further increased [13–15]. However, the rate of consistent condom use during anal intercourse in the past six months among Chinese MSM has been found to be still low (only 44.8%) [16]. Previous studies have shown that factors associated with CAI among MSM in China included older age, multiple male sexual partners, drinking alcohol before sex, engagement in CVI, psychological health, social stigma, and low level of social support [17–20]. However, limited studies were conducted to explore the association between CAI and HIV-related interventional services, perception of HIV risk, psychosocial factors such as suicidal inclinations, childhood sexual abuse, or adult sexual violence among Chinese MSM. Therefore, the identification of more comprehensive factors significantly associated with CAI is required to improve current prevention programs for this population.

HIV testing is essential to most HIV prevention approaches, and considered an intervention in its own right [21, 22]. Through HIV testing and counseling, infected individuals can be identified as early as possible and they can be provided with greater access to early treatment. It may also lead to reduce HIV-related risky behaviors, thereby decreasing the spread of HIV [23]. Promoting HIV testing can thus be considered an effective strategy for HIV prevention and control. However, the global HIV testing rate has been found to be rather low, even in areas where HIV is highly prevalent. Specifically, among 32 sub-Saharan African countries, the rate of HIV testing in the past year ranged from 1.6% in Niger to 41.7% in Eritrea [24]. In China, the annual HIV testing rate was 50.4% among MSM in 2011[25]. Several studies have examined HIV testing rate and determinants in China. For example, studies conducted among MSM in Beijing and Chongqing, China showed that the HIV testing rate in the recent year was 69% and 44.6%, respectively [26, 27]. Fear of being tested as HIV positive and perceiving no risk for HIV are two of critical reasons of not routinely testing for HIV specifically among Chinese MSM [26]. Previous studies indicated that certain factors, including multiple male sexual partners, knowing someone infected with HIV, disclosure of HIV status to male partners, and lower level of HIV-related stigma and discrimination were associated with recent HIV testing [26, 27]. However, limited studies were conducted to explore the association between recent HIV testing and receipt of HIV-related interventional services, such as those providing knowledge regarding antiretroviral therapy (ART) for HIV/AIDS, and receiving AIDS/STI interventions in Chinese MSM. Thus, it would be necessary to understand the characteristics of MSM who have recently tested for HIV and identify more significant and practical correlates of HIV testing in order to provide better targets for HIV prevention programs.

MSM have been the target population for HIV prevention in Zhejiang Province, China, in recent years; despite this, very few studies have been conducted to examine sexual risk behaviors and HIV testing among MSM in Zhejiang. With an area of 16,596 km^2^ and a population of approximately 9.018 million, Hangzhou City is the capital of and largest city in Zhejiang Province. The HIV prevalence among MSM in Hangzhou was 8.71% in 2011, and increased to 12.3% in 2013 [28]. This study aimed to determine the prevalence of sexual risk behaviors (e.g., CAI) and recent HIV testing rate among MSM in Hangzhou City and identify associated factors. We comprehensively examined sociodemographic characteristics, risky behaviors, socio-psychological factors, HIV risk perception, and HIV-related interventional services and testing history to identify the determinants of CAI and HIV testing rate. The results of the study can provide guidance for the implementation of future HIV intervention programs.

## Materials and methods

### Study population and sampling

This cross-sectional study was conducted in Hangzhou City, China, between December 2013 and June 2014. The study was part of fieldwork conducted in Hangzhou for a baseline survey in a technical research project aimed at controlling new infections of HIV/AIDS among MSM in Zhejiang Province, China. The study procedures and sampling strategies have been described in detail elsewhere [29]. In brief, participants were recruited using respondent-driven sampling (RDS) method, which is an approximate probability sampling method introduced by Heckathorn to recruit hard-to-reach human populations such as injection drug users and MSM [30]. MSM samples recruited via RDS are considered more diverse and representative of the population compared to those obtained via time-location sampling or snowball sampling [31, 32]. Participants who were at least 14 years old, had resided in Hangzhou for at least 3 months, self-reported having anal or oral sex in the past 12 months, and were willing to participate in the survey were considered to meet the inclusion criteria for this study.

Using RDS procedures, eight initial eligible participants served as seeds. “Seed” participants were purposively selected based on a diverse set of characteristics (age, socioeconomic status, other sociodemographics and linkages to other MSM) from the local MSM population. Then seed participants were asked to recruit partners or peers to participate in the survey; among them, one seed failed to recruit any individuals. After the seed participants completed the study questionnaires and had blood specimens drawn, they were provided with 3 recruitment coupons that acted as incentives. Specifically, in addition to receiving remuneration of 100 RMB (approximately 16 US$) for their participation in the study, participants received another 100 RMB for referring eligible peers to participate in this survey including completing a survey and blood drawing. Using incentives, the seed participants then recruited a maximum of 3 peer MSM from their social network to participate in this survey, and each of which in turn were asked to recruit up to three additional peers to participate in the study. This process was repeated until equilibrium was reached. More specifically, the sample composition was considered to have reached equilibrium when their socio-demographic characteristics were stable and would not change much for further recruitment of participants (the key sociodemographic characteristics such as age, marital status, educational level, monthly income, and sexual orientation changed < 2% in a subsequent wave recruitment of peers compared with the previous one), irrespective of the characteristics of the initial seeds. Ultimately, the seven seeds recruited 54, 62, 60, 133, 54, 50, and 90 MSM with a total sample of 511 MSM through 11 waves of recruitment.

Questionnaires used to collect data regarding sociodemographics, sexual risk behaviors, psychosocial conditions, HIV risk perception, and HIV-related interventional services and testing history were completed through face-to-face interviews with eligible participants, conducted by trained staff from the Centers for Disease Control and Prevention (CDC) of Xiacheng District (Detailed survey questionnaires in Chinese and English are provided in S1 and S2 Tables). Of the 511 MSM recruited, 459 (89.8%) had engaged in anal intercourse in the 6 months prior to the study; of these 459 participants, 457 (99.6%) answered the question regarding whether they had undergone an HIV test in the past year. Only the data of these 457 MSM were analyzed for recent HIV testing rate and determinants in the study.

### Measures

Sociodemographic characteristics included age, marital status, educational level, monthly income, insurance status, and sexual orientation. Sexual risk behaviors mainly were evaluated using the following data: number of male sexual partners within the 6 months preceding the survey (two or more sexual partners was defined as “multiple sexual partners”); engagement in anal intercourse with a male partner in the past 6 months; engagement in oral sex with a male partner in the past 6 months; engagement in vaginal intercourse in the past 6 months; and inconsistent condom use during anal, oral, or vaginal sex in the past 6 months. Psychosocial factors included experience of childhood sexual abuse, violence from an adult male sexual partner, and suicidal inclinations. Perception of HIV risk was assessed using two items: self-perceived likelihood of HIV infection and estimated HIV prevalence among MSM. Participants’ use of HIV-related interventional services included frequency of receiving AIDS/STI interventions in the past year, degree of awareness of antiretroviral therapy (ART) for HIV/AIDS, and HIV testing history in their lifetime and in the previous year.

The primary outcomes of interest in the analysis were CAI in the past 6 months (defined as affirming their engagement in inconsistent condom use with male partners during anal intercourse in the past 6 months prior to the survey) and recent HIV testing (defined as affirming the use of HIV testing in the recent year prior to the survey). All questionnaires were completed at the Love Working Group of Zhejiang, a gay outreach organization in Xiacheng District.

### Testing of HIV and syphilis

A venous blood specimen (5 ml) was drawn from each participant following the interview. The HIV screening test was performed by using enzyme-linked immunoassay (ELISA; Anti-HIV ELISA Kit, Zhuhai Livzon Diagnostics Inc., China), and if the result was positive, the specimen was retested using another ELISA kit (Anti-HIV ELISA Kit, Beijing Wantai Biological Pharmacy Enterprise Co. Ltd., China). If the results of one or both ELISA tests were positive, a western blot assay (HIV Blot 2.2, MP Diagnostics, Singapore) was conducted to confirm the diagnosis.

The syphilis test was conducted using another ELISA test kit (Diagnostic Kit for Antibody to Treponema Pallidum, Zhuhai Livzon Diagnostics Inc., China), and seropositive results were confirmed using a Toluidine Red Unheated Serum Test (Beijing Wantai Biological Pharmacy Enterprise Co. Ltd., China). All blood specimens were drawn and tested at the Xiaotianzhu Community Health Center.

### Statistical analysis

Data were entered into EpiData version 3.1 (http://www.epidata.dk/) via double entry. After the data were cleaned up and verified, statistical analysis was performed using PASW Statistics 18.0 (SPSS Inc., Chicago, IL, USA). For descriptive analyses, we calculated the mean, standard deviation (SD), median and interquartile range (IQR) for continuous variables, and frequencies and percentages for categorical variables. To calculate the RDS-weighted population estimates and bootstrapped confidence intervals (CIs) for all relevant variables, we used the Respondent-Driven Sampling Analysis Tool (RDSAT) version 7.1 (http://www.respondentdrivensampling.org/) to calculate by adjusting network size for data [30, 33, 34]. The algorithm type in RDSAT was set to “enhanced data-smoothing.” RDS-weighted estimates (with 95% CI) of the participants’ socio-demographic traits, HIV risk perception, psychosocial factors, HIV-related interventional services, and CAI, CVI, HIV testing, HIV and syphilis prevalence were computed in RDSAT version 7.1 by selecting “Analyze Partition” from the Analyze menu and selecting “Analyze” (Table 1). Stratified estimates of CAI and HIV testing rate for specific subgroups were computed in RDSAT (Tables 2 and 3).

**Table 1:** Crude and weighted estimates of characteristics of MSM in Hangzhou, China (N = 459).

Variables	Crude rate % (n/N)	RDS-weighted rate % (95% CI)
***Socio-demographics***		
**Age (years) (median = 30; IQR = 25–39; mean = 32.39; SD = 9.92)**		
**≤24**	23.5 (108/459)	24.8 (17.7–32.0)
**25–34**	41.2 (189/459)	41.0 (32.9–51.1)
**35–44**	22.2 (102/459)	22.0 (15.2–28.7)
**≥45**	13.1 (60/459)	12.2 (7.3–17.3)
**Marital status**^**1**^		
**Never married**	64.4 (295/458)	63.9 (53.8–71.0)
**Married/Divorced/Widowed**	35.6 (163/458)	36.1 (29.0–46.2)
**Educational level**^**1**^		
**Junior school or lower**	35.6 (163/458)	34.8 (27.5–45.4)
**High school**	25.5 (117/458)	24.5 (17.9–33.0)
**College or higher**	38.9 (178/458)	40.7 (30.0–48.4)
**Monthly income (RMB)**		
**<3000**	22.0 (101/459)	31.0 (21.8–38.6)
**3000–3999**	29.4 (135/459)	26.0 (19.4–34.4)
**≥4000**	48.6 (223/459)	43.1 (34.6–52.8)
**Insurance status**		
**Uninsured**	24.4 (112/459)	27.1 (19.7–36.7)
**Insured**	75.6 (347/459)	72.9 (63.3–80.3)
**Sexual orientation**^**1**^		
**Homosexual**	48.7 (223/458)	47.1 (37.9–55.4)
**Heterosexual, bisexual, or unknown**	51.3 (235/458)	52.9 (44.6–62.1)
***Sexual risk behaviors***		
**Age of first homosexual sex (median = 23; IQR = 20–28; mean = 24.75; SD = 7.69)**		
**≤19**	21.6 (99/459)	21.3 (13.8–27.1)
**20–29**	56.9 (261/459)	53.9 (45.8–63.5)
**≥30**	21.6 (99/459)	24.7 (18.3–33.0)
**Number of male sex partners for AI during the past 6 months**		
**1**	42.0 (193/459)	59.3 (50.8–67.2)
**2**	26.6 (122/459)	23.6 (17.4–30.8)
**>2**	31.4 (144/459)	17.1 (12.2–22.2)
**CAI with male partners in the past 6 months**		
**No**	54.9 (252/459)	56.3 (48.5–66.0)
**Yes**	45.1 (207/459)	43.7 (34.0–51.5)
**Had oral sex in the past 6 months**		
**Yes**	90.6 (416/459)	89.3 (83.4–93.7)
**No**	9.4 (43/459)	10.7 (6.3–16.6)
**Condom use during oral sex in the past 6 months**		
**Always**	1.0 (4/416)	0.3 (0.1–0.5)
**Not always**	99.0 (412/416)	99.7 (99.5–99.9)
**Had vaginal sex with a woman in the past 6 months**^**9**^		
**Yes**	28.0 (126/450)	30.0 (23.6–40.6)
**No**	72.0 (324/450)	70.0 (59.4–76.4)
**CVI in the past 6 months**		
**No**	6.5 (30/459)	7.6 (4.3–12.6)
**Yes**	20.9 (96/459)	21.6 (15.6–32.3)
***Psychosocial factors***		
**Suicidal inclinations**^**1**^		
**No**	92.6 (424/458)	92.8 (87.7–96.5)
**Yes**	7.4 (34/458)	7.2 (3.5–12.3)
**Experienced violence from male sexual partners**^**1**^		
**No**	98.9 (453/458)	99.1 (97.9–99.9)
**Yes**	1.1 (5/458)	0.9 (0.1–3.1)
**Experienced sexual abuse in childhood**^**1**^		
**Yes**	6.3 (29/458)	4.4 (2.1–6.2)
**No**	93.7 (429/458)	95.6 (93.8–97.9)
***Risk perception***		
**Self-perceived likelihood of HIV infection**^**6**^		
**Probable**	5.7 (26/453)	5.1 (1.6–10.9)
**Possible**	12.4 (56/453)	11.4 (6.8–17.2)
**Unlikely**	33.8 (153/453)	32.2 (25.0–43.6)
**Impossible**	48.1 (218/453)	51.3 (39.6–58.0)
**Estimated HIV prevalence among MSM**^**2**^		
**≤5%**	19.3 (88/457)	16.5 (10.0–21.3)
**6–10%**	22.1 (101/457)	18.6 (13.7–27.5)
**≥11%**	58.6 (268/457)	64.9 (56.8–72.5)
***HIV-related interventional services***		
**Knew about ART for HIV/AIDS**^**7**^		
**Much**	17.3 (78/452)	12.2 (8.2–17.3)
**A little**	14.6 (66/452)	18.8 (11.4–26.4)
**Not at all**	68.1 (308/452)	69.0 (60.6–77.1)
**Frequency of receiving AIDS/STI interventions in the past year**^**1**^		
**Never**	43.9 (201/458)	50.5 (42.4–60.1)
**Once or twice**	48.7 (223/458)	46.0 (36.7–54.1)
**More than twice**	7.4 (34/458)	3.6 (1.7–5.6)
**HIV testing in the recent year**^**2**^		
**Yes**	62.8 (287/457)	56.8 (48.7–66.5)
**No**	37.2 (170/457)	43.2 (33.5–51.3)
***HIV and syphilis tests***		
**HIV status**		
**Positive**	13.1 (60/459)	8.8 (5.6–13.0)
**Negative**	86.9 (399/459)	91.2 (87.0–94.4)
**Syphilis infection**		
**Positive**	8.5 (39/459)	6.5 (2.7–12.2)
**Negative**	91.5 (420/459)	93.5 (87.8–97.3)
**Co-infection of HIV and syphilis**		
**Positive**	2.8 (13/459)	2.0 (0.6–3.8)
**Negative**	97.2 (446/459)	98.0 (96.2–99.4)

Note: CI: confidence interval; MSM: men who have sex with men; AI: anal intercourse; CAI: condomless anal intercourse; CVI: condomless vaginal intercourse; ART: antiretroviral therapy; STI: sexually transmitted infection; IQR: interquartile range; HIV: human immunodeficiency virus; AIDS: acquired immunodeficiency syndrome; RDS: respondent-driven sampling; Footnote 1, 2, 6, 7, and 9 indicates the number of people missing this information.

**Table 2:** Factors associated with CAI in the past 6 months (N = 459).

Variable	Crude rate %(n/N)	RDS-weighted rate % (95% CI)	OR (95% CI)	AOR (95% CI)
***Socio-demographics***				
**Age (years)**				
**≤24**	52.8 (57/108)	57.1 (40.3–70.9)	1.44 (0.77–2.69)	0.85(0.36–2.03)
**25–34**	43.4 (82/189)	36.4 (23.0–51.6)	0.58 (0.32–1.05)	0.46(0.22–1.00)
**35–44**	39.2 (40/102)	35.8 (18.7–49.1)	0.64 (0.34–1.24)	0.69(0.34–1.41)
**≥45**	46.7 (28/60)	52.2 (34.8–75.0)	1	1
**Marital status**				
**Never married**	46.4 (137/295)	45.2 (34.6–56.9)	1.17 (0.80–1.73)	0.48(0.26–0.90)
**Married/Divorced/Widowed**	42.9 (70/163)	41.3 (25.8–51.5)	1	1
**Education level**				
**High school or lower**	42.1 (118/280)	41.3 (29.6–50.0)	1	1
**College or higher**	50.0 (89/178)	47.5 (32.3–61.6)	1.28 (0.88–1.86)	1.12(0.69–1.83)
**Monthly income (RMB)**				
**<4000**	45.3 (107/236)	46.8 (34.2–57.2)	1.51 (1.04–2.20)	1.46(0.90–2.35)
**≥4000**	44.8 (100/223)	38.4 (25.0–49.6)	1	1
**Sexual orientation**				
**Homosexual**	45.3 (101/223)	50.4 (36.7–64.5)	1.67 (1.15–2.42)	1.48(0.94–2.34)
**Heterosexual, bisexual, or unknown**	45.1 (106/235)	37.8 (26.3–47.0)	1	1
***Sexual risk behaviors***				
**Age of first homosexual sex**				
**≤19**	54.5 (54/99)	60.3 (41.1–74.4)	2.48 (1.43–4.32)	3.31 (1.34–8.15)
**20–29**	43.3 (113/261)	40.2 (28.1–51.0)	1.15 (0.73–1.82)	1.90 (0.95–3.81)
**≥30**	40.4 (40/99)	35.6 (21.1–52.3)	1	1
**Number of male sex partners for AI in the past 6 months**				
**≤2**	42.5 (134/315)	42.0 (31.1–51.2)	1	
**>2**	50.7 (73/144)	50.2 (36.1–65.7)	1.31 (0.80–2.13)	
**Had oral sex in the past 6 months**				
**Yes**	46.9 (195/416)	43.9 (34.0–52.1)	1.07 (0.58–1.96)	
**No**	27.9 (12/43)	42.7 (15.7–64.6)	1	
**Had vaginal sex with a woman in the past 6 months**				
**Yes**	42.1 (53/126)	42.3 (25.8–54.9)	0.78 (0.52–1.18)	
**No**	46.6 (151/324)	45.4 (33.9–54.9)	1	
**CVI in the past 6 months**				
**No**	26.7(8/30)	49.7(2.0–65.7)	1	
**Yes**	46.9(45/96)	41.5(3.5–57.2)	1.10(0.51–2.40)	
***Psychosocial factors***				
**Suicidal inclinations**				
**No**	43.4 (184/424)	42.2 (31.9–49.5)	1	1
**Yes**	67.6 (23/34)	65.0 (34.4–89.2)	3.03 (1.40–6.57)	1.99 (1.03–4.78)
**Experienced violence from male sexual partners**				
**No**	45.3 (205/453)	44.0 (34.1–51.9)	1	
**Yes**	40.0 (2/5)	18.2 (10.4–30.5)	0.16 (0.01–2.87)	
**Experienced sexual abuse in childhood**				
**Yes**	75.9 (22/29)	74.5 (59.6–98.0)	3.11 (1.19–8.13)	3.05 (1.02–9.15)
**No**	43.1 (185/429)	42.3 (31.7–49.9)	1	1
***Risk perception***				
**Self-perceived likelihood of HIV infection**				
**Probable**	61.5 (16/26)	31.4 (10.3–81.9)	1	
**Possible/unlikely/impossible**	44.0 (188/427)	44.4 (34.5–52.4)	1.73 (0.69–4.33)	
**Estimated HIV prevalence among MSM**				
**≤5%**	58.0 (51/88)	61.7 (42.8–79.3)	2.52 (1.50–4.21)	1.89 (1.00–3.55)
**6–10%**	47.5 (48/101)	48.3 (26.9–64.3)	1.35 (0.83–2.19)	1.37 (0.79–2.36)
**≥11%**	40.3 (108/268)	38.2 (27.3–49.3)	1	1
***HIV- related interventional services***				
**Knew about ART for HIV/AIDS**				
**Much**	55.1 (43/78)	55.8 (39.2–73.5)	1	1
**A little**	48.5 (32/66)	41.5 (21.9–67.6)	0.56 (0.28–1.13)	0.70(0.31–1.58)
**Not at all**	41.2 (127/308)	38.1 (26.5–46.3)	0.43 (0.24–0.79)	0.61(0.31–1.20)
**Underwent AIDS/STI interventions in the past year**				
**No**	50.7 (102/201)	42.9 (28.9–54.7)	1	
**Yes**	40.9 (105/257)	43.9 (31.1–54.0)	1.05 (0.72–1.51)	
**HIV testing in the recent year**				
**Yes**	42.5 (122/287)	46.8 (35.4–56.9)	1.28 (0.88–1.86)	1.55(1.00–2.41)
**No**	50.0 (85/170)	41.3 (26.9–54.4)	1	1
***HIV and syphilis test***				
**HIV infection**				
**Positive**	46.7(28/60)	51.0(29.0–69.8)	1.42(0.73–2.77)	
**Negative**	44.9(179/399)	43.5(33.0–52.0)	1	
**Syphilis infection**				
**Positive**	41.0(16/39)	27.8(9.5–62.3)	0.49(0.21–1.16)	0.63(0.25–1.60)
**Negative**	45.5(191/420)	45.1(34.5–52.9)	1	1

Note: OR: odds ratio; AOR: adjusted odds ratio; CI: confidence interval; MSM: men who have sex with men; AI: anal intercourse; CAI: condomless anal intercourse; CVI: condomless vaginal intercourse; ART: antiretroviral therapy; STI: sexually transmitted infection; HIV: human immunodeficiency virus; AIDS: acquired immunodeficiency syndrome; RDS: respondent-driven sampling.

**Table 3:** Factors associated with recent HIV testing among MSM (N = 457).

Variable	Crude rate % (n/N)	RDS-weighted rate % (95% CI)	OR (95% CI)	AOR (95% CI)
***Socio-demographics***				
**Age(years)**				
**≤24**	60.2 (65/108)	48.0 (31.0–61.1)	0.70 (0.37–1.32)	2.26(0.59–8.65)
**25–34**	61.7 (116/188)	59.4 (49.2–77.5)	1.30 (0.72–2.34)	8.48(2.50–28.81)
**35–44**	66.3 (67/101)	61.9 (44.8–79.8)	1.19 (0.62–2.28)	3.86(1.11–13.39)
**≥45**	65.0 (39/60)	55.3 (35.9–79.8)	1	1
**Marital status**				
**Never married**	62.9 (185/294)	54.9 (43.3–67.8)	1	1
**Married/Divorced/Widowed**	62.3 (101/162)	60.5 (50.2–75.1)	1.44 (0.97–2.13)	1.37(0.44–4.30)
**Education level**				
**High school or lower**	65.6 (183/279)	57.6 (47.4–69.7)	1	1
**College or higher**	58.2 (103/177)	56.2 (41.7–70.8)	1.07 (0.74–1.56)	1.49(0.66–3.39)
**Monthly income (RMB)**				
**<4000**	64.5 (151/234)	56.2 (44.1–67.8)	1	1
**≥4000**	61.0 (136/223)	57.9 (44.4–72.1)	1.10 (0.76–1.60)	0.57(0.26–1.25)
**Sexual orientation**				
**Homosexual**	67.6 (150/222)	54.7 (41.9–69.7)	0.81 (0.56–1.17)	0.47(0.22–1.01)
**Heterosexual, bisexual, or unknown**	58.5 (137/234)	59.1 (48.6–70.2)	1	1
***Sexual risk behaviors***				
**Age of first homosexual sex**				
**≤19**	57.6 (57/99)	41.6 (25.1–60.0)	0.50 (0.29–0.87)	0.14 (0.03–0.69)
**20–29**	62.3 (162/260)	61.1 (50.0–74.5)	1.05 (0.67–1.65)	0.40 (0.13–1.06)
**≥30**	69.4 (68/98)	58.8 (40.9–73.8)	1	1
**Number of male sex partners for AI in the past 6 months**				
**≤2**	67.4 (211/313)	58.4 (49.0–69.4)	1	1
**>2**	52.8 (76/144)	49.3 (33.9–63.6)	0.69 (0.43–1.13)	0.88(0.37–2.11)
**CAI with male partners in the past 6 months**				
**No**	66.0 (165/250)	54.8 (43.0–67.7)	1	1
**Yes**	58.9 (122/207)	60. 3(49.0–71.9)	1.28 (0.88–1.87)	2.75(1.32–5.75)
**Had oral sex in the past 6 months**				
**Yes**	61.4 (254/414)	54.3 (45.1–64.3)	0.33 (0.16–0.68)	0.30(0.09–1.00)
**No**	76.7 (33/43)	78.9 (60.7–93.6)	1	1
**Had vaginal sex with a woman in the past 6 months**				
**Yes**	61.6 (77/125)	62.1 (46.4–76.3)	1.19 (0.79–1.80)	
**No**	62.8 (203/323)	56.7 (47.7–68.4)	1	
**CVI in the past 6 months**				
**No**	63.3 (19/30)	83.8 (89.9–97.9)	1	
**Yes**	61.1 (58/95)	49.6 (43.7–92.8)	0.60 (0.27–1.35)	
***Psychosocial factors***				
**Suicidal inclination**				
**No**	63.0 (284/451)	57.7 (49.1–67.5)	1	1
**Yes**	58.8 (20/34)	42.8 (21.5–83.2)	0.54 (0.26–1.13)	0.89(0.17–4.56)
**Experienced violence from male sexual partners**				
**No**	63.0 (284/451)	57.2 (49.2–66.5)	1	1
**Yes**	40.0 (2/5)	3.3 (0.1–10.2)	0.05 (0.001–2.02)	0.32(0.01–40.33)
**Experienced sexual abuse in childhood**				
**Yes**	62.1 (18/29)	49.7 (34.1–83.5)	0.75 (0.31–1.85)	
**No**	62.8 (268/427)	57.1 (48.6–66.9)	1	
***Risk perception***				
**Self-perceived likelihood of HIV infection**				
**Probable**	76.9 (20/26)	86.2 (50.2–97.8)	4.41 (1.37–14.16)	6.49(1.03–41.00)
**Possible/unlikely/Impossible**	62.4 (265/425)	54.5 (46.4–64.8)	1	1
**Estimated HIV prevalence among MSM**				
**≤5%**	54.5 (48/88)	44.1 (25.3–62.4)	1	1
**6–10%**	57.4 (58/101)	51.6 (34.7–75.5)	1.36 (0.73–2.52)	0.95(0.28–3.24)
**≥11%**	67.3 (179/266)	61.1 (50.7–73.0)	2.01 (1.20–3.34)	0.81(0.28–2.34)
***HIV-related interventional services***				
**Knew about ART for HIV/AIDS**				
**Much**	83.3 (65/78)	76.4 (55.4–91.7)	3.28 (1.64–6.56)	7.27 (2.20–23.98)
**A little**	63.6 (42/66)	57.3 (40.2–78.3)	1.26 (0.77–2.06)	1.62 (0.61–4.33)
**Not at all**	57.5 (176/306)	51.6 (42.2–63.7)	1	1
**Underwent AIDS/STI interventions in the recent year**				
**No**	21.0 (42/200)	20.9 (11.4–31.9)	1	1
**Yes**	95.3 (244/256)	93.2 (87.6–98.5)	55.11 (29.94–101.44)	186.33 (72.36–479.81)
***HIV and syphilis test***				
**HIV infection**				
**Positive**	60.3(35/58)	48.3(28.5–72.1)	0.67(0.34–1.34)	
**Negative**	63.2(252/399)	57.7(48.8–68.0)	1	
**Syphilis infection**				
**Positive**	69.2(27/39)	78.6(48.9–93.6)	3.16(1.23–8.16)	12.27(3.22–46.72)
**Negative**	62.2(260/418)	54.4(45.6–64.4)	1	1

Note: OR: odds ratio; AOR: adjusted odds ratio; CI: confidence interval; MSM: men who have sex with men; AI: anal intercourse; CAI: condomless anal intercourse; CVI: condomless vaginal intercourse; ART: antiretroviral therapy; STI: sexually transmitted infection; HIV: human immunodeficiency virus; AIDS: acquired immunodeficiency syndrome; RDS: respondent-driven sampling

The individualized weights produced in RDSAT were imported into PASW Statistics 18.0 to conduct weighted logistic regression analysis. To determine whether multicollinearity was present in the variable set, we examined the variance inflation factors. Variables that were associated with the outcome variables at *P* ≤ 0.20 in the bivariate logistic regression model were included in a multivariate logistic regression model controlling main demographic variables (age, educational level, marital status, monthly income, and sexual orientation), wherein we calculated adjusted odds ratios (AORs) and 95% CIs. The goodness of fit of the model was evaluated using the Hosmer—Lemeshow test. The statistical significance threshold was *P* < 0.05.

### Ethics statement

The study was approved by the ethical review board of Zhejiang Provincial Center for Disease Control and Prevention. Participants were not harmed by their participation in the study and their data remained confidential. Written informed consent was signed by all participants during the survey. Participants with positive results for the HIV or syphilis test were informed and counseled by the staff of Xiacheng District CDC and received the necessary referral services. This informing and counseling was provided at the same location as the interviews (i.e., Love Working Group of Zhejiang).

## Results

### Sample characteristics

The ages of the 459 participants ranged from 17 to 65 years (mean = 32.39, SD = 9.92; median = 30, IQR = 25–39). According to the weighted estimates, 24.8% of participants were aged 17–24 years and 63.9% had never been married. Furthermore, 40.7% of participants had an educational level of college or higher and roughly one quarter (27.1%) had no insurance plan. Approximately 31.0% of participants had a monthly income of less than 3000 RMB, and about half (47.1%) of the MSM reported their sexual orientation as homosexual or gay (Table 1).

### Description of sexual risk behaviors and HIV testing rate

Of the 459 participants, the age of first homosexual sex ranged from 12 to 63 years (mean = 24.75, SD = 7.69; median = 23, IQR = 20–28). The weighted estimates revealed that 21.3% reported a homosexual debut at 12–19 years old, while 40.7% reported having multiple (≥ 2) male anal intercourse partners during the past 6 months. The RDS-weighted rate of CAI with male partners during the past 6 months was 43.7% (95% CI, 34.0–51.5%). Furthermore, 89.3% of participants had engaged in oral sex during the past 6 months, of whom 99.7% had engaged in unprotected oral sex. An estimated 30.0% had engaged in vaginal intercourse during the past 6 months, among whom the rate of CVI was 21.6% (95% CI, 15.6–32.3%). The RDS-weighted rate of recent HIV testing was 56.8% (95% CI, 48.7–66.5%).

### HIV and syphilis testing

The prevalence rates of HIV and syphilis were 13.1% and 8.5% among 459 MSM, respectively, while the RDS-weighted rates were 8.8% (95% CI, 5.6–13.0%) and 6.5% (95% CI, 2.7–12.2%). The prevalence of co-infection of HIV and syphilis was 2.8%, with a weighted prevalence of 2.0% (95% CI, 0.6–3.8%).

### Factors associated with CAI in the past 6 months

Table 2 shows the results of the bivariate and multivariate logistic regression analysis of factors associated with CAI in the past 6 months. Younger age at homosexual debut, suicidal inclinations, childhood sexual abuse, lower estimates of HIV prevalence among MSM, and not knowing about ART for HIV/AIDS were associated with CAI in the bivariate analysis (Table 2). In the multivariate analysis, CAI was associated with younger age at homosexual debut, with higher odds of CAI for participants with a debut at 12–19 years of age relative to those participants with a debut at ≥ 30 years of age (AOR = 3.31, 95% CI, 1.34–8.15); suicidal inclinations (AOR = 1.99; 95% CI, 1.03–4.78); childhood sexual abuse (AOR: 3.05, 95% CI: 1.02–9.15); HIV testing in the recent year (AOR:1.55, 95% CI: 1.00–2.41); lower estimate of HIV prevalence among MSM, with higher odds of CAI for participants who estimated it to be lower than 6% than those who estimated it to be higher than 10% (AOR = 1.89, 95% CI, 1.00–3.55).

### Factors associated with recent HIV testing

Table 3 shows the results of the bivariate and multivariate analysis of the correlates of HIV testing in the recent year. The results of the bivariate analysis indicated that age at homosexual debut, having oral sex in the past 6 months, self-perceived higher likelihood of HIV infection, higher estimates of HIV prevalence among MSM, knowing about ART for HIV/AIDS, receiving AIDS/STI interventions in the past year, and syphilis infection were associated with HIV testing in the past year (Table 3). The multivariate analysis, by contrast, revealed that HIV testing in the recent year was associated with knowing about ART for HIV/AIDS, with higher odds of HIV testing for participants who knew much about ART than those who knew nothing (AOR = 7.27, 95% CI, 2.20–23.98). CAI in the past 6 months (AOR = 2.75; 95% CI, 1.32–5.75), self-perceived higher likelihood of HIV infection (AOR = 6.49; 95% CI, 1.03–41.00), receiving AIDS/STI interventions in the past year (AOR = 186.33; 95% CI, 72.36–479.81) and syphilis infection (AOR = 12.27; 95% CI, 3.22–46.72) were associated with HIV testing in the previous year. Notably, MSM who reported a homosexual debut at 12–19 years of age were less likely to have undergone a recent HIV test relative to those who reported a homosexual debut at ≥ 30 years of age (AOR = 0.14, 95% CI, 0.03–0.69), and those who had engaged in oral sex in the past 6 months were less likely to have undergone recent HIV testing (AOR = 0.30; 95% CI, 0.09–1.00).

## Discussion

The study examined the prevalence of sexual risk behaviors (e.g., CAI and CVI) and recent HIV testing, and associated factors among MSM in Hangzhou, China using a relatively representative RDS sample. In this study, 43.7% of participants reported engaging in CAI in the previous 6 months and 40.7% had engaged in anal intercourse with at least two male sex partners. This CAI rate is consistent with reports of low consistent condom use among MSM in other cities in China—for example, in Wenzhou, prevalence of CAI in the past 6 months with a man was found to be around 58.1% [35], while among a sample of 642 MSM in Changsha, 28.7% reported having CAI with male partners at their last sexual activity [17]. In addition, only 43% of MSM reported having consistently used condoms during anal sex in the past half year in the whole country in 2011 [25]. Moreover, a similar study conducted in Nanjing, China among a RDS sample of 430 MSM also confirmed the higher prevalence of CAI (62.3%) in Nanjing, China [18]. However, in contrast to the previous study, our study also showed that more than one third of the MSM had ever been married, approximately one third of MSM had had vaginal sex with a woman during the past 6 months, and that the rate of consistent condom use during such sex was very low (23.3%). This implies that the population of MSM could potentially act as a bridge to transmit HIV to the general female population, which indicates that increased attention should be paid to high-risk behaviors among men who have sex with both men and women (MSMW).

About half of MSM (56.8%) had undergone HIV testing in the recent year prior to the survey. This rate was found to be somewhat higher than the national annual HIV testing rate (50.4%) among Chinese MSM in 2011 [25]. However, the annual testing rate among these Hangzhou MSM was also far below the rate reported in many developed countries, such as 67% in the USA in 2011 [36] and 72.4% in Australia in 2014 [37]. However, in another study, the rate for HIV testing in the recent year for MSM was considerably higher relative to that observed for female sex workers (17.7%) [38]. Most (86.2%) HIV-positive MSM were unaware of their infection at the time of the survey. This is consistent with the result reported by Chow [39], who used a mathematical model and estimated that 87% of all Chinese HIV-infected MSM remained undiagnosed. A previous study indicated that people who were unaware that they were infected with HIV were 3.5 times as likely to transmit HIV as diagnosed people [40].

Taken together, high prevalence of HIV and syphilis, high rates of CAI and CVI, low testing rate, and high number of undiagnosed HIV-infected MSM indicates a high risk of HIV infection and transmission in this population. In addition, the finding that HIV prevalence was considerably higher, relative to syphilis prevalence, in this sample was both interesting and unexpected. A similar finding was reported in a previous study involving MSM in Guangzhou. The reason for these findings could be that the high HIV testing rate could have aided in controlling syphilis infection, because MSM who undergo HIV testing are offered free syphilis screening and referral services for treatment and behavioral interventions [41]. The results also showed a strong association between HIV testing and syphilis infection. This suggests that increasing HIV testing coverage as an intervention not only contributed to the reduction of new HIV infection but also aided in controlling syphilis infection.

About one fifth of MSM had a homosexual debut at the age of 12–19 years in the study. MSM who reported younger age at homosexual debut were more likely to engage in CAI and less likely to undergo recent HIV testing. Similarly, a study conducted in Switzerland revealed that younger age at participants’ first anal intercourse experience was associated with greater likelihood of unprotected intercourse [42]. Thus, knowing the age of first homosexual intercourse and the context in which it occurs is important in the development of preventive interventions [43], particularly since such behavior appears to be an important correlate of sexual risk behaviors such as CAI and avoidance of HIV testing, in addition to the risk of HIV and STI acquisition. Furthermore, it is preferable to provide such intervention measures early, prior to individual’s first sexual experience, to prevent engagement in behaviors that place them at greater risk of HIV infection. Both knowledge- and skill-based interventions designed to address the needs of MSM must be comprehensive and multifaceted. Moreover, preventive interventions for HIV/AIDS should account for first sexual experiences (both context and partners), specifically targeting those who report a homosexual debut before the age of 19 years. In addition, schools could establish sexual educational classes and interesting activities to help young men build healthy relationships during their school years. Creating comprehensive, youth centered, culturally appropriate sex education, healthy and safe sexual activities, and content specific to their need lessons should be considered for any sex education or HIV prevention programs, which aim to decrease CAI or HIV among young MSM.

In the current study, MSM with psychosocial problems, such as suicidal tendencies and childhood sexual abuse, were more likely to engage in CAI. A study conducted in Shanghai showed that increased suicidal ideation was associated with involuntary subordination and engagement in risky sex [44]. A longitudinal study conducted in 6 cities in the USA also revealed that MSM with experience of childhood sexual abuse were more likely to engage in CAI and were at greater risk of HIV acquisition [45]. Childhood sexual abuse has been found to predict depression and tendencies toward self-destructive behavior, such as self-harm and suicidal attempts in both men and women [46]. Previous studies have shown that social support from peers and partners, family support, and the provision of counseling to reduce stress and improve behavioral self-management skills, were effective in reducing rates of CAI and intercourse with multiple partners among MSM [47–50]. Such interventions, particularly those designed to improve family and social support, could be beneficial to Chinese MSM. Indeed, highly comprehensive interventions that target psychological, behavioral, and social aspects are required to address existing mental health problems, with special attention paid to MSM with a history of childhood sexual abuse and suicidality. Health providers and MSM community organizations are valuable in providing social and emotional support to alleviate mental disorder, and promote healthy behaviors among MSM. Besides, family members and friends from outside the MSM community should also offer social resources and support to help MSM to mitigate psychosocial health problems.

More than half of MSM did not know about the existence of ART in the study, which points to a possible lack of awareness of available treatment for HIV among this group in China. The results also showed that knowledge of ART for HIV/AIDS was positively associated with recent HIV testing. This finding is consistent with that of a study conducted in three cities in Mozambique [24]. However, another reason could be that getting more HIV tests was associated with increased accompanying counseling, during which ART information was disclosed. This suggests that efforts to prevent HIV/AIDS among MSM are relatively insufficient at present in China. Knowledge of the benefits of frequent HIV testing, such as extending the life span and reducing likelihood of transmission by accepting therapy in time [51, 52], might motivate more MSM to undergo HIV testing in China. Thus, the finding suggests the need to increase the number of existing HIV/AIDS educational programs targeting MSM.

Lower estimate of HIV prevalence among MSM was found to be a factor influencing CAI in our study. Promoting local MSM to understand high HIV prevalence among them might help these individuals realize the presence of HIV-infected individuals around them and may help improve their awareness of high risk of HIV acquisition and its relation to unsafe sex. Thus, we might consider adding the local HIV prevalence to HIV prevention and educational programs targeting MSM in China. In our study, only 5.1% of MSM perceived their high risk of HIV acquisition, and perceiving higher risk was strongly associated with HIV testing while actually the HIV prevalence was 8.8% among MSM in Hangzhou in this study. CAI in the past 6 months was associated with HIV testing in the past 12 months in the study; however, our study indicates MSM engaging in oral sex were less likely to test for HIV. These findings imply that the current knowledge about HIV infection risk among Chinese MSM is insufficient and improving their risk perception is very important for HIV prevention.

The AIDS/STI interventions examined in the study included those involving condom and lubrication provision, STI examination and treatment, distribution of educational material and AIDS/STI information, which could improve HIV testing rate among MSM. Compared to MSM who had not received AIDS/STI interventions in the recent year, those who had received such interventions were more likely to have undergone an HIV test. The result suggests that the frequency with which AIDS/STI interventions are implemented should be increased and expanded in future prevention for HIV.

There are several limitations in our study. First, the sample size was modest; therefore, some of the nonsignificant associations observed could have occurred because of the inadequate power. However, we adopted the RDS method to increase the representativeness of our sample. Second, because the data were collected via self-report of MSM participants, it may involve reporting bias (i.e., social desirability and recall bias); the true prevalence of some sensitive variables such as sexual risk behaviors, suicidality, and childhood sexual abuse could have been underestimated in the rather homophobic and hostile environment in China. Third, we cannot infer any causality in the associations between CAI and recent HIV testing and other factors due to the cross-sectional design of the study. Fourth, our study was conducted in Hangzhou, and the sample did not represent populations from the other areas in China. Finally, the main purpose of the original survey was to determine HIV prevalence and examine associated factors, and we did not consider certain variables known to be associated with CAI and recent HIV testing, such as social support, HIV/sexual stigma, and discrimination. Therefore, longitudinal studies involving large sample sizes and a greater variety of psychosocial and behavioral measures are required to confirm our findings.

## Conclusions

In summary, Chinese MSM tend to have a high HIV prevalence far higher than syphilis, high amounts of CAI and CVI, and a low HIV testing rate, all of which constitute a high risk of HIV infection and transmission. HIV prevention interventions that aim to reduce CAI and scale-up HIV testing must be directed towards MSM, particularly those with an early homosexual sexual debut and psychosocial health problems. Furthermore, it is necessary to increase the number of HIV/AIDS educational programs that target topics such as the benefits of ART and the estimated HIV prevalence among MSM and improve risk perception of HIV acquisition.

## Supporting information

S1 Table Health questionnaire for MSM (in Chinese). (DOCX)

S2 Table Health questionnaire for MSM (in English). (DOCX)
