# Supplementary material for: Prevalence of condomless anal intercourse and recent HIV testing and their associated factors among men who have sex with men in Hangzhou, China: A respondent-driven sampling survey
Source: PLoS One. 2017 Mar 8;12(3):e0167730. doi: 10.1371/journal.pone.0167730 (PMC5342181; doi:10.1371/journal.pone.0167730)
Supplement: S2 Table — (DOCX) [file pone.0167730.s002.docx]

ID code：

（Paste ID code ）

Coupon code：

**Health questionnaire for men who have sex with men (MSM)**

Name or alia of participant:

Investigated city：

Investigation agency：

November,2013

**Participant inclusion and exclusion**

| **S1** | Birthday：___month ___year | | |
| --- | --- | --- | --- |
|  | **S11** | If age≥14 years old？**①**Yes **②**No**(not included in this survey)** |  |
| **S2** | Recent resident address：___Prefecture(District) ___City，resident time ___year(s)**（less than 1 year is converted into decimal）** | | |
|  | **S21** | If you reside in recent place ≥3 months？**①**Yes **②**No**(not included in this survey)** |  |
| **S3** | If you have oral or anal sex with male sexual partner(s) in the past year **①**Yes **②**No **(not included in this survey)** | |  |

**A. Basic information**

| **A1** | Marital status：①Cohabitation(with female) ②Cohabitation(with male) ③Single ④Married with spouse Divorced or windowed |  |
| --- | --- | --- |
| **A2** | Educational level:  **①**Primary school or below **②**Junior school **③**High school or second school **④**College or above |  |
| **A3** | Occupation：   1. Employee（Restaurant/hotel/beauty salon/property logistics and other industries）**②**Company employees **③**Businessman **④**Government agency/institution staff **⑤**Worker（Construction/manufacturing, etc）**⑥**Student **⑦**Farmer **⑧**Retired **⑨**Unemployed **⑩**Others（note） |  |
| **A4** | Monthly income(RMB: yuan)？   1. No income **②**<1000 **③**1000-1999 **④**2000-2999 **⑤**3000-3999 **⑥**≥4000 |  |
| **A5** | Medical insurance（Single or multiple choices）？  ①[New](javascript:void(0);) [Rural](javascript:void(0);) [Co-operative](javascript:void(0);) [Medical](javascript:void(0);) [System](javascript:void(0);)②The medical insurance for urban employees③The [medical](javascript:void(0);) [insurance](javascript:void(0);) [for](javascript:void(0);) [urban](javascript:void(0);) [residents](javascript:void(0);) ④The commercial insurance ⑤No |  |
| **A6** | Self-reported sex orientation？   1. Homosexual ②Heterosexual ③Bisexual ④Indeterminate |  |
| **A7** | Venues to seek for sexual partners:   1. Bars, dance halls, tea room, club ②Bath, sauna, foot massage, massage ③Park, public toilets, the grass ④Internet **⑤**Others（note） |  |
| **A8** | Locally, how many MSM do you know about in your circle of friends_____?**[Knowing about means you will be able to know the other face, know the name or alias, nicknames, and contact information, and can be in touch with the other in a month. Give some time to the participants for thinking carefully]** | |
| **A9** | The relationship with the male who recommended you to participate in this program:   1. The past sexual partners**（before 6 months）②**The sexual partners in the past 6 months **③**Good friends   **④**Common friends **⑤**Acquaintances **⑥**Strangers |  |
| **A10** | Self-perceived status of physical health about yourself？**①**Very well **②**Well **③**Normal **④**Not well **⑤**Very bad |  |

**B. Sexual behavioral characteristics and sexual partner’ network**

| **B1** | Age of sexual debut with a man: ___years old | | | | | | | |
| --- | --- | --- | --- | --- | --- | --- | --- | --- |
| **B2** | Number of **male** sexual partners with you with **anal intercourse** in the past **6** months________**（If there is no, jump to B4）** | | | | | | | |
| **B3** | Frequency of condom usage engaging in **anal sex** with **male** sexual partners in the past **6** months？ | | | | | | | |
|  | **B31** | Anal intercourse with regular male sexual partners：**①**Never **②**Sometimes **③**Always **④**There was no such sexual activity | | | | | |  |
|  | **B32** | Anal intercourse with casual male sexual partners：**（Neither commercial or regular）**：**①**Never **②**Sometimes **③**Always **④**There was no such sexual activity | | | | | |  |
|  | **B33** | Anal intercourse with male sex workers：**①**Never **②**Sometimes **③**Always **④**There was no such sexual activity | | | | | |  |
|  | **B34** | Group anal intercourse：**①**Never **②**Sometimes **③**Always **④**There was no such sexual activity | | | | | |  |
|  | **B35** | Anal intercourse after drinking wine or beer: **①**Never **②**Sometimes **③**Always **④**There was no such sexual activity | | | | | |  |
|  | **B36** | Anal intercourse after taking drugs：**①**Never **②**Sometimes **③**Always **④**There was no such sexual activity | | | | | |  |
| **B4** | Number of **male** sexual partners with you with **oral sex** in the past **6** months**（If there is no, jump to B6）** | | | | | | | |
| **B5** | Frequency of condom usage engaging in **oral sex** with **male** sexual partners in the past **6** months？ | | | | | | | |
|  | **B51** | Oral sex with regular male sexual partners：**①**Never **②**Sometimes **③**Always **④**There was no such sexual activity | | | | | |  |
|  | **B52** | Oral sex with casual male sexual partners：**（Neither commercial or regular）**：**①**Never **②**Sometimes **③**Always **④**There was no such sexual activity | | | | | |  |
|  | **B53** | Oral sex with male sex workers：**①**Never **②**Sometimes **③**Always **④**There was no such sexual activity | | | | | |  |
| **B6** | Please recall the male sexual partner in the recent 1 year one by one , you don't need to tell their names, but in order to help you recall this information, you can give them the number or use code (**Please according to frequency of sex from more to less listed in sequence, less than 5 people, all listed; more than 5 people, fill in only five people**) | | | | | | | |
|  | **Sexual partner’ characteristics** | | **No.1** | **No.2** | **No.3** | **No.4** | **No.5** | |
|  | **B61** | **Relationship between you and male sexual partners：**  **①** Regular male sexual partners **②**Casual male sexual partners**（Neither commercial nor regular）③** Commercial male sexual behavior  **④**Other___ |  |  |  |  |  | |
|  | **B62** | **Age of the sexual partner(years)：①**<20 **②**20～**③**30～**④**40～**⑤**≥50 |  |  |  |  |  | |
|  | **B63** | **Frequency of sexual behavior：**  **①**≥twice every week **②**4～7times every month **③**Once to 3 times every month **④**<once every month |  |  |  |  |  | |
|  | **B64** | **HIV-positive status of sexual partners：①**Positive **②**Negative **③**Unclear |  |  |  |  |  | |
| **B7** | Age of sexual debut with a woman:**①**years old **②**There was no sex with women**（Jump to C1）** | | | | | | |  |
| **B8** | Number of **female** sexual partners with you with **sex** in the past **6** months________**（If there is no, jump to C1）** | | | | | | | |
| **B9** | Frequency of condom usage engaging in **anal sex** with **female** sexual partners in the past **6** months？ | | | | | | | |
|  | **B91** | Spouse**(regular)**female sexual partners: **①**Never **②**Sometimes **③**Always **④**There was no such sexual activity | | | | | |  |
|  | **B92** | Casual female sexual partners （**Neither commercial or regular**）：**①**Never **②**Sometimes **③**Always **④**There was no such sexual activity | | | | | |  |
|  | **B93** | Female sex workers：**①**Never **②**Sometimes **③**Always **④**There was no such sexual activity | | | | | |  |

**C. Measurement of mood（****CES-D）**

| The following is your possible feelings or acts, pointed out that all kinds of feelings and behavior occurrence in  the last week according to your actual situation, please fill in the corresponding number：   1. Rarely or none of the time (<1 day) ②Some or a little of the time (1-2 days) 2. Occasionally or a moderate amount of the time (3-4 days) ④Most or all of the time (5-7 days) | | |
| --- | --- | --- |
| **C1** | I was bothered by things that don’t usually bother me. |  |
| **C2** | I did not feel like eating; my appetite was poor. |  |
| **C3** | I felt that I could not shake off the blues even with the help of my family or friends. |  |
| **C4** | I felt that I was just as good as other people. |  |
| **C5** | I had trouble keeping my mind on what I was doing. |  |
| **C6** | I felt depressed. |  |
| **C7** | I felt everything I did was an effort. |  |
| **C8** | I felt hopeful about the future. |  |
| **C9** | I thought my life had been a failure. |  |
| **C10** | I felt fearful. |  |
| **C11** | My sleep was restless. |  |
| **C12** | I was happy. |  |
| **C13** | I talked less than usual. |  |
| **C14** | I felt lonely. |  |
| **C15** | People were unfriendly. |  |
| **C16** | I enjoyed life. |  |
| **C17** | I had crying spells. |  |
| **C18** | I felt sad. |  |
| **C19** | I felt that people disliked me. |  |
| **C20** | I could not get “going”. |  |

**D. Behavior and social cultural characteristics**

| **D1** | Do you smoke? **[Smoking means smoking one or more cigarettes every day for more than a year or smoking more than 300 cigarettes in the short term (3 months or less**)**]**   1. Current smoker **②**Former smoker**(Having never smoked in the past 6 months)**   **③**Never smoke **(Jump to D2)** | |  |
| --- | --- | --- | --- |
|  | **D11** | When did you start to smoke？Age_____ (or _____year).  Accumulated ____years of smoking excluding time not to smoke up to now. | |
|  | **D12** | **Number of cigarettes you smoke on average every day. (Generally a pack of cigarettes is 20 cigarettes.)________** | |
| **D2** | Do you drink**[Drinking refers to drinking alcohol intake of 100 grams per week (2 liang), such as 250 grams of 40 degrees wine consumption, 1 kg of 10 degrees wine intake]**   1. Drinking now **②**Drinking before **(No drinking in the past six months) ③**Never drinking**(Jump to D5)** | |  |
|  | **D21** | From ____aged years old (or ___years), you started to smoke.  Accumulated ____years of drinking excluding time not to drink up to now. | |
|  | **D22** | Times of drinking a week on average(drinking intake drink every time generally)：   1. Beer___ bottles(___ml) **②**Red Winebottles（ml）**③**White spirit ___liang（___degree wine）   **④**Yellow wine ___liang（degree wine）**⑤**Others，wine**(What kind)** ___ml（___degree wine） | |
| **D3** | If you were drunk in the past year? **①**Often **②**Sometimes **③**Never**(Jump to D5)** | |  |
| **D4** | If you want or need to reduce your drink in the past 1 year? **①**Yes **②**No | |  |
| **D5** | If you have more than amount of drug use in the past 1 year(**including Psychotropic drugs, such as sedative hypnotics, caffeine, wheat carlin, LSD, and narcotic drugs, such as opiates, cocaine, cannabinoids, etc**)?   1. Often **②**Sometimes **③**Never**(Jump to D7)** | |  |
| **D6** | If you want or need to reduce your drug use? **①**Yes **②**No | |  |
| **D7** | Have you ever suicide tendency？**①**Never **②**Sometimes **③**Often | |  |
| **D8** | If you suffered from a male sexual partner violence?（**violence includes physical, sexual or mental violence**）   1. Never **②**Sometimes **③**Often | |  |
| **D9** | If you suffered from sexual abuse in your childhood?**①**Yes **②**No | |  |
| **D10** | What impact do you think the current social perception about MSM will have on sexual behavior?   1. Increasing protected sex with a male**②**Increasing protected sex with a female **③**Increasing unprotected sex with a male **④**Increasing unprotected sex with a female **⑤**No impact | |  |
| **D11** | Self-perceived possibility of HIV infection: **①**Probable **②**Possible **③**Unlikely **④**Impossible | |  |
| **D12** | Estimate of HIV prevalence among MSM now: **①**≤5% **②**6-10% **③**11-15% **④**16-20% **⑤**≥21% | |  |
| **D13** | **Frequency of receiving HIV/STI interventions in the past year（**including giving out a condom/lubricant or publicity materials,, venereal disease inspection or treatment, AIDS/STD consulting, the training of AIDS/STD knowledge prevention, etc**）**   1. Never **②**1~2 times a year **③**2~5 times half year **④**1~3 times a month **⑤**1~4times a month | |  |

**E. The history and intention of HIV testing**

| **E1** | Have you ever undertaken an HIV testing?**①**Yes **②**No**（Jump to E6）** | |  |
| --- | --- | --- | --- |
| **E2** | Times of HIV testing in the past year:__________ | | |
| **E3** | Date of the recent HIV testing: ___month ___ year | | |
| **E4** | The type of the recent HIV testing:   1. VCT (Voluntary Counseling And Testing) **②**Testing service provided by CDC **③**Blood transfusion (blood products) **④**Blood donation **⑤**Physical examination for employment   **⑥**Premarital physical examination **⑦**Hospitalized examination **⑧**Physical examination for enrollment **⑨**Other（Please specify） | |  |
| **E5** | Do you have the habit of regular HIV testing？**①**Yes **②**No**（Jump to E6）** | |  |
|  | **E51** | The reason of regular HIV testing**（Single or multiple choices）**:   1. Health Surveillance **②**Frequent high-risk sexual behaviors **③** Distrusting the regular sexual partner **④**The regular sexual partner is positive **⑤** To start a new partnership with the other **⑥**Other（Please specify） |  |
|  | **E52** | How long do you make a HIV test?（**Jump to F1 after finishing the question）**   1. 3 months **②**Half year **③**One year **④**Other（Please specify） |  |
| **E6** | Are you willing to accept regular testing for HIV? **①**Yes**（Jump to F1）②**No | |  |
|  | **E61** | If no, the reason is______（**Single or multiple choices）**   1. Don't know where to test ②It is difficult to take a test on a regular time due to work or the living habits ③Worry about the positive test results   **④**It is not convenient to go to the test place **⑤**Fear of the discrimination after their identity exposure **⑥**There is no high-risk behavior  ⑦It is very safe for regular sexual partners **⑧**Other（Please specify） |  |

**F. Cognition antiviral treatment and intention of early treatment**

| The following is the relevant information of HIV/AIDS antiviral treatment, please read carefully.  Benefits from early treatment:  1．The faster CD4 cells level rises, the lower the risk of death is.  2．The better the immune function recovery is, the smaller the [probability](javascript:void(0);) of opportunistic infections is.  3．The slower virus reproduction in the body, the smaller body organs are damaged.  4．The better the physical condition, the better to the resistant to drug side effects.  5．The faster the viral load slows down, the smaller the risk of infection of spouse or sexual partners is.  **Note of treatment:**  1. Treatment first need patients to have a strong intention and psychologically prepare to take lifelong medication.  2. Resistance to medicines is mostly because of poor medication compliance, but most people can take medicines regularly.  3. You may feel discomfort when taking medicine at first, generally to relieve in 2 to 6 weeks.  4. Drug side effects vary from person to person, and regular monitoring can early detect and resort to the doctor for help. | | |
| --- | --- | --- |
| **F1** | Did you know about antiviral treatment for HIV/AIDS? **①**Very well **②**Generally **③**A little **④**Almost no |  |
| **F2** | On antiviral treatment, you may be more attention to what aspects (**multiple choices if possible**)?   1. Medicine effect ②Medicine side effect ③[Medication](javascript:void(0);) [compliance](javascript:void(0);)   ④ Privacy protection **⑤**Convenience of taking medicine **⑥**Resistance to drugs  **⑦**Financial burden **⑧**Other（Please specify） |  |
| **F3** | What do you think it is necessary to accept early antiviral treatment for HIV-positive people? **①**Yes **②**No |  |
| **F4** | Which of the following suggestions do you think can most promote HIV-positive people to accept the early  antiviral treatment?   1. Doctor ② CDC worker **③**Group volunteer **④**Patient **⑤**Other（Please specify） |  |

**This questionnaire is now over! Thank you for your participation! Welcome to put forward your precious suggestion for the survey!**

**­**

**The following results is completed by the investigator**

| **G1** | Syphilis testing result：the first method：①Positive ②Negative |  |
| --- | --- | --- |
| **G2** | Syphilis testing result：the second method：①Positive ②Negative |  |
| **People infected with HIV before (not including positive people for HIV testing in this survey), just fill out the G6-G8.** | | |
| **G3** | At the beginning, the screening method for HIV:________ |  |
| **G4** | HIV screening result： ①Positive ②Negative**（Finishing this survey）** |  |
| **G5** | HIV confirmed result：①Positive ②Negative**（Finishing this survey）** |  |
| **G6** | Card number for outbreak： |  |
| **G7** | Viral load results for HIV testing：Date for viral load testing：___mm/dd/yyyy | |
| **G8** | CD4 result：Date for CD4 testing：___mm/dd/yyyy | |

**­­­**

**Please make sure whether you have completed the following processes:**

1. **Participant inclusion and exclusion**②**Informed consent ③Omission items reviewing after completing questionnaire**

**Investigator： Investigation Date：_______(**mm/dd/yyyy)
